# Design and Protection Strategy of Distributed Intrusion Detection System in Big Data Environment

**DOI:** 10.1155/2022/4720169

**Published:** 2022-06-29

**Authors:** Rong Chen

**Affiliations:** Shanghai Customs College, Shanghai 201204, China

## Abstract

One of the important research topics is protecting the host from threats by developing a reliable and accurate intrusion detection system. However, since the amount of data has grown fast due to the emergence of big data, the performance of traditional systems designed to identify breaches has suffered several flaws. One of them, for example, is known as single-point failure; low adaptability and a high false alarm rate are also typical. Hadoop is used to detect intrusions to tackle these difficulties. The Java system is used to create a framework with a significant data flow that detects intrusions when a distributed system is built. The proposed solution employs a distributed operating system for data collection, storage, and analysis. The results indicate that external distributed denial of service (DDoS) attacks are recognized quickly. The single-point failure issue is overcome, alleviating the bottleneck problem of data processing ability.

## 1. Introduction

More complicated and costly security issues appear to be treated efficiently as computer network technology rapidly advances. Hence, they trigger to cause the development of diverse technologies that deal with information security in several aspects. For example, while the firewall generally defends information security as an intrusion detection technology, the upgraded version is called a defense firewall [1, 2]. The intrusion detection system aims typically at collecting and crunching critical information and utilizes some predefined rules or protocols to detect irregular actions or transitions occurring supposedly against security policies and the predefined set of rules that were constructed using historical records when network technology is under investigation. By doing so, unauthorized, abnormal, or out-of-predefined rules are timely alerted to the security unit of the system. The system's efficiency is related to an issue called the single-point failure that the intrusion detection system faces often. Besides, the implementation constraints of processing vast and complicated data under the significant data cases grow more complex [3, 4]. Therefore, a novel approach called intrusion detection systems employing distributed systems under a big data environment could bring plenty of advantages for protecting information security.

Big data brings several issues to deal with, namely, a vast data set whose structure changes fast and includes diverse data types such as numeric, categoric, and unstructured. Besides, the standard computer configuration processing mode cannot satisfy the different requirements to process big data. So, Hadoop, launched by the Apache community [5] as a platform to crunch big data, employs a mapping reduction model (MapReduce) to conduct fundamental tasks of sorting and calculations. Moreover, Hadoop provides a cluster architecture to distribute tasks to the constituents of the distributed system. Hadoop functions as independent clusters, so the whole system continues to operate when any failure occurs regarding one or several nodes. A distributed computing proficiency is associated with the cluster architecture of Hadoop. In other terms, when each node of a cluster is assigned to take care of large-scale data analysis tasks, the efficiency of data analysis is greatly improved. Therefore, this capability can be smoothly implemented to detect intrusions. In conclusion, the issues related to single-point failure and bottlenecks due to the low capacity of data processing can be effectively resolved.

Parallel programs featuring distributed architecture can be written, run, executed, and employed to run calculations regarding massive data sets on Hadoop clusters, an open-source distributed parallel programming widely implemented by enterprises and research institutions. MapReduce, the computing model of Hadoop, constructed by Google, is an effective tool.

The Hadoop distributed file system (HDFS) uses two key technologies. While the first one is the storage tool, the other is called the MapReduce distributed computing framework. The storage mechanism provides the underlying support for Hadoop. HDFS generally consists of Client, Datanode, and Namenode. When a cluster utilizes the architecture of Hadoop, a host called the Namenode and several hosts called the Datanode are available. The client represents the program employing HDFS. Namenode is a responsible host in the Hadoop cluster to save information of data nodes and distribute computational tasks and the final protocol. Datanode is responsible for data storage and processing. To ensure data security, HDFS increases the amount of redundant data moderately. The specific method is to save three copies of the same data in different Datanodes.

The parallel computing process running on a large-scale cluster is split into two functions: Map and Reduce. The other key technology is called MapReduce distributed computing framework, a mode to process and generate large datasets. The calculation process of MapReduce is based on decomposing large datasets into thousands of small datasets. Then, some datasets are distributed to a cluster node to process and produce intermediate outcomes. Finally, these results are obtained by several nodes to form the final outputs.

Big data technology is combined with the process of intrusion detection in [6], and an extensible quasi-real-time intrusion detection system employing Hadoop is suggested, which uses Hive and Mahout technologies to detect p2p botnet attacks. The Hive module functions as surveilling and processing network traces. Since the Mahout module provides parallel solid processing capability, constructing a decision tree model of random forest can be smoothly realized to detect botnets. A distributed intrusion detection system based on cloud computing utilizing the K-means clustering algorithm is proposed by [7], which resulted in higher detection accuracy. An unsupervised method to reduce dimensionality issues that combine t-SNE and a hierarchical neural network is suggested to detect the behavior of attacks [8]. By doing so, mapping high-dimensional network data space is shrunk into low-dimensional space. A method based on employing a distributed ensemble learning to cope with a misbehavior-aware on-demand collaborative intrusion detection system is proposed in [9], whose advantage is to reduce the number of life threats and road congestions caused by network attacks on VANET.

However, the research to cope with intrusion detection systems concerning significant data implementation issues has been at a developmental stage, so there is no longer a full-fledged method. Therefore, the research direction is on the track of improving the conventional ones toward the ones satisfying the necessities of big data.

The motivation of this research is summarized two-fold: increasing detection accuracy and adaptability to big data implementations by taking care of the single-point failure problems when the available distributed intrusion detection system model is a concern. Thus, a distributed intrusion detection system dealing with big data is proposed. In this effort, Hadoop's cluster computing environment [10] and its operational storage features are employed to utilize the Amazon DynamoDB database and Java architecture to design more robust intrusion detection components.

The rest of the manuscript is structured as follows: Section 2 presents the aspects of data such as data collector, transceiver, data analysis, and data-based alert systems.  Section 3 is allocated to both results and discussion.  Section 4 concludes the research by stressing the advantages of the proposed method.

## 2. The Proposed Method

Big data architecture is intended to manage data input, processing, and analysis that are too massive or complicated for typical database systems. The threshold at which businesses join the extensive data domain varies depending on the users' and tools' capabilities. It might imply hundreds of gigabytes of data for some and hundreds of terabytes for others. The definition of big data evolves as technologies for dealing with large datasets improve. This word increasingly refers to the value you can extract from your data sets using sophisticated analytics rather than the amount of the data, which tends to be pretty huge in many circumstances. The data landscape has evolved. What you can and are expected to do with data have shifted. Storage costs have dropped substantially, but the methods for collecting data have expanded. Some data comes at a quick speed, requiring ongoing collection and observation. Other data comes more slowly but in massive quantities, often in the form of decades of historical data. You might be dealing with advanced analytics or a cyber security dilemma. These are the problems that big data architectures aims to overcome.

**Figure 1:**
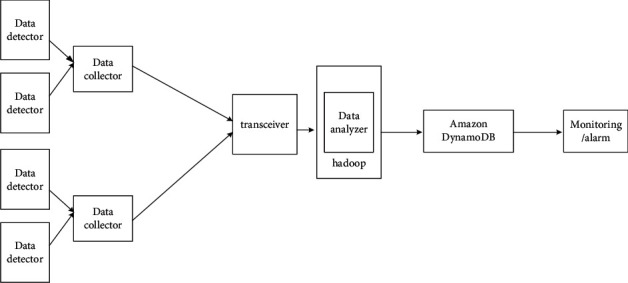
The structure diagram of the distributed intrusion detection system.

Figure 1 depicts the logical components that comprise the proposed big data architecture. Its architecture has features: a data detector, data collector, agent, transceiver, and data analysis center.

It must be noted that there is not another comparable model to use as a pilot. Consequently, to avoid bias or incorrect impressions, the paper presents the performance of the proposed model in an innovative dataset. The experiments were made exclusively for this research approach and presented only in this paper. Every element in this design is included in the suggested individual solution, as discussed in the following sections.

### 2.1. The Detection of Data

A system coping with acquiring data and analyzing events as a unit located at the bottom of the system is called a data detector (DD). DDs are categorized into two types, host-based and network-based DDs, per different data resources. For general indexes such as central processing unit (CPU), memcachd (MEM) utilization, transmission control protocol (TCP) connections [11], and network bandwidth, a DD employs a capture service to determine indexes with a minimum interval length, then sends data to middleware, and finally returns it to the data center. For different indexes such as user behavior logs and WEB servers running records on other computers, a DD regularly utilizes a log monitoring service to capture the latest log information. Moreover, the system has no restrictions on the DD [12].

### 2.2. Data Collector

The DD is unique to each monitoring host. The data acquisition agent controls all local DDs. When the DD is in charge of transmitting data to the transceiver, it needs to send data to the agent and then the agent transmits it to the transceiver. The Chukwa of Apache software is employed as the data collector [13], which detects the contents of files written by DDs. If a new range exists in the file, it will be input into the collector of Chukwa following the specific rules and the collection will be input into Amazon DynamoDB [14].

The monitored host's CPU utilization, memory, TCP connections, and network bandwidth are collected. The DD, written in Java, employs SNMP-V3 based on the TCP convention family to handle the dispatching line. Then, the DD establishes a connection with the SNMP service through the SNMP driver package [4]. The reference is made to port 161 and obtains information by transmitting an analogous SNMP monitoring ID to the server. The workflow of the DD is composed of four sections. First, the DD is initialized and then linked to the SNMP service of the monitored machine [15]. Second, the data detector traverses each node in the SNMP service tree according to the OID set by the developer, and the stop condition is utilized to find the node with the same settings. Third, different threads are opened in the DD, and finally, each line grabs the data according to the minimum level and writes the captured data into the development file.

### 2.3. Data Transceiver and Middleware

Data transceiver divides the system monitoring network into multiple areas, and each site is composed of a group of transceivers and numerous data collectors. The operation of the data transceiver will sort out and process the received data [16]. A single-point failure may occur at one of these areas corresponding to a transceiver. Therefore, the transceiver adopts a redundancy strategy, and multiple transceivers can coexist in one area. When the data acquisition agent transmits data, it randomly selects a transceiver to transfer the data, which helps efficiently balance the available load [17]. The data middleware functions to cluster new information and loosen up the data analysis center to investigate information in the data. For this purpose, a message queue called RocketMQ is employed as the data receiving and sending middleware in this manuscript.

### 2.4. Data Analysis Center

The distributed intrusion detection system is constructed by the mode of “distributed detection and storage, and centralized analysis.” While the monitoring host stores some intrusion or suspicious data at the bottom of the system locally and hoards the rest in the data analysis center, it utilizes the characteristics of suspicious events to pinpoint the intrusion behaviors that cannot be detected otherwise.

The Hadoop cluster framework, whose two types of nodes have specific roles, is implemented [18]. While one is called the common computing node, the other is called the task submission node. The task submission node will periodically submit data to analyze tasks in a cluster. Then, the corresponding process is described as follows: the number of partitioned tasks is firstly computed based on data size. Then, each task is partitioned and distributed to each cluster node to make calculations. If the calculation task determines specification steps, the partition outcomes of each task will be summarized, calculated, and presented. Otherwise, the results will be provided as output directly. The Hadoop framework deals with the whole process by allocating and scheduling tasks and recovering errors. Thus, users only define computational tasks of MapReduce, processing methods used for data fragmentation, and specification methods.

The proposed protocol has an intriguing quirk: the reducers never directly communicate with one another, but only through the mappers in the following round. MapReduce handles grouping and message passing, along with engineering challenges like fault tolerance or load balancing, which are all controlled by MapReduce. The proposed mathematical protocol's most significant suggestion is to encode a limit on the total amount of space consumed. Specifically, the algorithm takes a key-value pair list as input:

(1) $$\begin{array}{c} k_{i},v_{i,i=1}^{N}. \end{array}$$

As a whole size,

(2) $$\begin{array}{c} n=\sum_{i=1}^{N}\left|k_{i}\right|+\left|v_{i}\right|. \end{array}$$

The mapper *m* is a Turing machine that takes a single key-value pair *k, v* as input and returns a list of key-value pairs as output:

(3) $$\begin{array}{c} k_{1}^{\prime },v_{1}^{\prime },\ldots ,k_{s}^{\prime },v_{s}^{\prime }. \end{array}$$

If MRC[*f*(*n*), *g*(*n*)] is the round bound and the second argument is the time bound, the logarithmic number of rounds is defined as

(4) $$\begin{array}{c} {\text{MRC}}^{i}=\text{MRC}\left[\log^{i}\left(n\right),\text{poly}\left(n\right)\right]. \end{array}$$

On the other hand, the mapper *ρ* is a Turing machine that takes a single key-value *k* as input and a list of values *v*_1_,…, *v*_*m*_ and produces a new list as *v*_1_′,…, *v*_*M*_′.

Each reducer determines what the finishing state would be if the *G* is a graph that had started in state *s* after processing the chunk of the input for each conceivable state *s* in G. As a result, the output of reducer *j* would be an encoding of the following table:

(5) $$\begin{array}{c} \begin{array}{c} s_{1}⟶T_{j}\left(s_{1}\right),\\ s_{2}⟶T_{j}\left(s_{2}\right),\\ \vdots \\ s_{\left|S\right|}⟶T_{j}\left(s_{\left|S\right|}\right). \end{array} \end{array}$$

If

(6) $$\begin{array}{c} \text{MRC}\left[\text{poly}\left(n\right),1\right]⊊\text{MRC}\left[\text{poly}\left(n\right),\text{poly}\left(n\right)\right], \end{array}$$

and

(7) $$\begin{array}{c} \text{MRC}\left[1,n\right]⊊\text{MRC}\left[n,n\right]\subset \text{MRC}\left[1,n^{2}\right]⊊\text{MRC}\left[n^{2},n^{2}\right]\subset \ldots . \end{array}$$

The process is

(8) $$\begin{array}{c} \text{MRC}\left[1,\text{poly}\left(n\right)\right]=\text{MRC}\left[\text{poly}\left(n\right),\text{poly}\left(n\right)\right]. \end{array}$$

The size (number of edges) and storage space needed in the computer memory to neighborhood list of a graph (number of nodes) are

(9) $$\begin{array}{c} \left(\overset{n}{\underset{i=1}{\sum }}\left(1+\deg\left(v_{i}\right)\right)\right)=O\left(n+m\right). \end{array}$$

We have

(10) $$\begin{array}{c} \deg\left(v_{i}\right)=d_{\text{out }}\left(v_{i}\right),1\leq i\leq n. \end{array}$$

Therefore, the total complexity required to implement the algorithm is

(11) $$\begin{array}{c} O\left(n\right)+\sum_{v\in V}O\left(\left|\text{Adj}\left(v\right)\right|\right)=O\left(n+m\right). \end{array}$$

The cost function we want to minimize during the reduction process is as follows:

(12) $$\begin{array}{c} \frac{1}{2}\left\|w\right\|+C\sum_{i=1}^{n}\log\left(\exp\left(-y_{i}\left(w^{T}x_{i}+b\right)\right)+1\right), \end{array}$$

where *C* > 0 and *b* are the coefficients representing the penalty of incorrect results.

So, to be able to estimate the probability distribution of the process, we limit the history to *n* processes:

(13) $$\begin{array}{c} P\left(w_{T}|w_{1},w_{2},\ldots ,w_{T-1}\right)\approx P\left(w_{T}|w_{T-n},\ldots ,w_{T-2},w_{T-1}\right). \end{array}$$

Through maximum likelihood estimation, we calculate

(14) $$\begin{array}{c} P\left(w_{3}|w_{1},w_{2}\right)=\frac{\text{count}\left(w_{1},w_{2},w_{3}\right)}{\sum_{w}\text{count}\left(w_{1},w_{2},w\right)}. \end{array}$$

Therefore,

(15) $$\begin{array}{c} x=\left[C\left(w_{1}\right);C\left(w_{2}\right);\ldots ;C\left(w_{n}\right)\right].\\ \hat{y}=P\left(w_{i}|w_{1:k}\right)=LM\left(w_{1:k}\right)=\text{softmax}\left(hW^{2}+b^{2}\right).\\ h=g\left(xW^{1}+b^{1}\right).\\ x=\left[C\left(w_{1}\right);C\left(w_{2}\right);\ldots ;C\left(w_{n}\right)\right].\\ C\left(w\right)=E_{\left[w\right]}.\\ \text{where }w_{i}\in V,E\in {\mathbb{R}}^{|V|\times d_{w}},W^{1}\in {\mathbb{R}}^{n\cdot d_{w}\times d_{hid\;}},b^{1}\in {\mathbb{R}}^{d_{\text{hid }}},W^{2}\in {\mathbb{R}}^{d_{\text{hid }}\times |V|},b^{2}\in {\mathbb{R}}^{|V|}.\; \end{array}$$

Finally, we propose a method that first linearly increases the learning rate and then decreases it linearly for reducers. The proposed algorithmic system is as follows:

(16) $$\begin{array}{c} \begin{array}{c} \text{cut}=\left[T\cdot \text{ cut\_frac}\right],\\ p=\left\{\begin{array}{cc} \frac{t}{\text{cut}}, & \text{if }t<\text{cut},\\ 1-\frac{t-\text{cut}}{\text{ cut}.\;\left(1/\text{ cut\_frac }-1\right)}, & \text{else}, \end{array}\right.\\ \eta_{t}=\eta_{\max}\cdot \frac{1+p\cdot \left(\text{ratio}-1\right)}{\text{ ratio }}. \end{array} \end{array}$$

The idea is to adapt the parameters to the characteristics of a particular set of processes. The model should first converge quickly to a suitable area of the parameter space and then improve the parameters.

### 2.5. Monitoring of the System and Alarming Service

The monitoring system mainly surveils the CPU, MEM, TCP connection, network bandwidth, and other fundamental sections of each host, as well as the running status of the monitoring host [19]. The external invasion trace can be determined during the process, and the corresponding measures can be taken when data detection is utilized [20]. The system has an alert facility located at the highest stage, and its function is to judge whether the system is within the normal operation bounds. The alarm service receives information from the data analysis [21] center and monitoring system. When there would be abnormal data [22] or an aberrated host running state, the alarm service would send an alarm to inform the administrator that the system could be under attack [23].

The spring MVC framework and velocity template technology are employed to implement the module to monitor the system. This module in charge of surveilling is composed of modules to allocate the management of a user, monitor the system, and manage Amazon DynamoDB [24].

The login page of the user management module accepts information in a template form. Whenever information is input to log in, the token must be verified [25]. A user verified as logged in by the token directly reaches the home page. In contrast, the receipt confirms the login information, which results in successful verification. Then, it will jump into the login interface directly. Otherwise, it will be directed to the error interface.

The monitoring module contains two functions: index view and index definition. While JavaScript and velocity technology implement the index view function, the index definition function is implemented by the JavaScript Hight Charts drawing function library. The Amazon DynamoDB management module is employed to manage online Amazon DynamoDB to reduce the management complexity [26].

## 3. Results and Discussion

**Figure 2:**
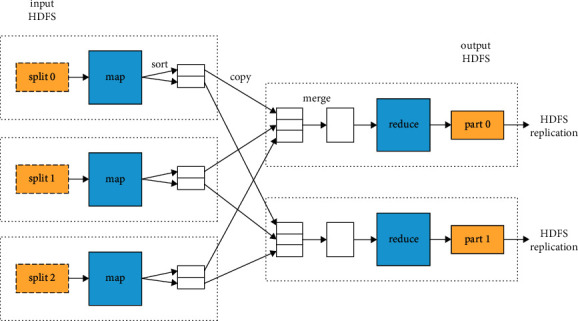
MapReduce processing.

**Figure 3:**
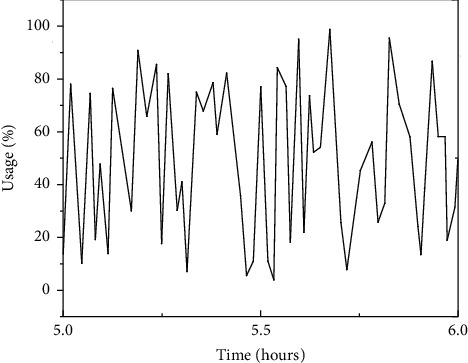
The figure plot showing the trend of the CPU index (Server 1).

The DD module in this manuscript's distributed intrusion detection system grabs data at the minimum level. The CPU utilization rate captured by the DD module was gauged between 0 : 00 to 6 : 00 am on December 5, to depict the operation condition of the CPU system, which is shown in Figures 2 and 3.

Currently, the main security problem in implementing big data services has been distributed denial of service (DDoS) attack, which is taken as the research object. It must be noted that while firewalls and intrusion prevention systems (IPS) are essential for network security, they are insufficient to guard against complicated DDoS assaults. Modern DDoS attack tactics need a multifaceted strategy that allows users to examine Internet infrastructure and network availability. Consider the following capabilities for improved DDoS protection and quicker mitigation of TCP SYN flood DDoS attacks:

1. Support for both inline and out-of-band deployment to guarantee the network has no single point of failure
2. It has broad network visibility, including the ability to observe and analyze traffic from many networks segments
3. Various threat information sources, such as statistical anomaly detection, customized threshold alerts, and fingerprints of known or new threats, ensure rapid and precise detection
4. There is scalability to handle assaults of various sizes, from low end (e.g., 1 Gbps) to high end (e.g., 10 Gbps and 40 Gbps)

The offense instrument of the DDoS is utilized to propose the distributed intrusion system.

**Figure 4:**
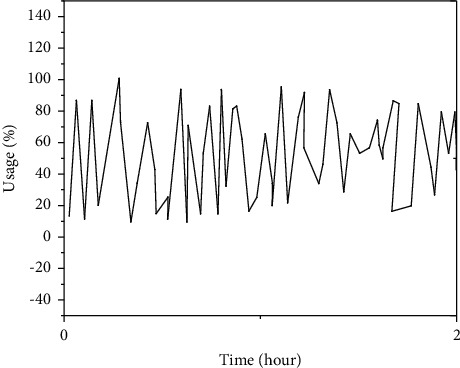
The figure plot showing the trend of the CPU index (Server 2).

Figure 4 depicts that the number of TCP connections fluctuates from 0 to 100 between 0 and 20 minutes, and the changing trend is found to be relatively stable, proving that the system has not received attacks.

**Figure 5:**
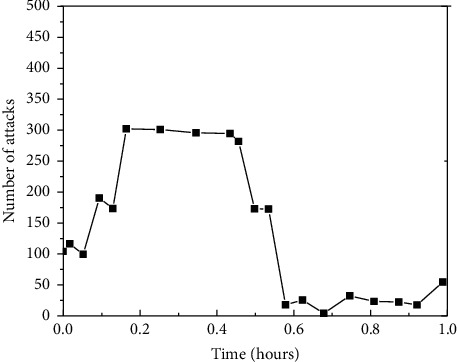
The figure plot depicts the changing trend of the CPU when the system is underattacked or invaded.

In Figure 5, between 20 through 40 minutes, the DDoS attacks are launched on the monitored system and the number of TCP connections increases to 300. Therefore, external attacks and intrusions can be detected through the monitoring system.

## 4. Conclusion

The Java system is employed to devise and execute a distributed intrusion detection framework by implementing the Hadoop framework when big data is considered. Thus, a novel protection method is put forward. The proposed method bringing advantages to the literature can be summarized as follows: (1) Distributed data acquisition, distributed processing, and distributed analysis are realized. (2) Monitoring CPU, MEM, and TCP indexes of the controlled host, external attacks, and intrusions are well detected. The corresponding alert services are provided when the DD collaborates with the data collector, transceiver middleware, and the center responsible for analyzing data.

The proposed method resolves the issues related to single-point failure and the low operating efficiency of the original distributed intrusion system. However, it still has some limitations. In the future research agenda, the combination with the machine learning algorithm would potentially contribute to the proposed intrusion detection methods, a capability that improves the ability of self-learning and adaptivity of the system. In conclusion, the most critical aspect will be reached by having both efficient and precise detection accuracy.

MapReduce is a programming framework, not an algorithm in and of itself, and complexity analysis is usually reserved for algorithms. But, a future expansion of the proposed approach will be the complexity analysis of MapReduce operations by getting the appropriate variables, for example,

(17) $$\begin{array}{c} O\left(n\log n*s*\left(\frac{1}{p}\right)\right), \end{array}$$

where *n* is the number of items, *s* is the number of nodes, and *p* is the ping time between nodes (assuming equal ping times between all nodes in the network).

## Data Availability

Data will be provided upon request to the author.

## References

[1] Gonzalez-Cuautle D., Hernandez-Suarez A., Sanchez-Perez G. (2020). Synthetic minority oversampling technique for optimizing classification tasks in botnet and intrusion-detection-system datasets. *Applied Sciences*.

[2] Li L., Yu Y., Bai S., Hou Y., Chen X. (2018). An effective two-step intrusion detection approach based on binary classification and $k$ -nn. *IEEE Access*.

[3] Gao Y., Chen X., Du X. (2020). A big data provenance model for data security supervision based on PROV-DM model. *IEEE Access*.

[4] Rathore M. M., Son H., Ahmad A., Paul A., Jeon G. (2018). Real-time big data stream processing using GPU with spark over Hadoop ecosystem. *International Journal of Parallel Programming*.

[5] Li M., Li R., Lee P. P. C. (2017). Relieving both storage and recovery burdens in big data clusters with R-STAIR codes. *IEEE Internet Computing*.

[6] Singh K., Guntuku S. C., Thakur A., Hota C. (2014). Big data analytics framework for peer-to-peer botnet detection using random forests. *Information Sciences*.

[7] Logesh K., Sumathi D. (2020). Journal of critical reviews K-means algorithm based distributed intrusion detection system for cloud computing environment. *Journal of Critical Reviews*.

[8] Yao H., Li C., Sun P. (2020). Using parametric t-distributed stochastic neighbor embedding combined with hierarchical neural network for network intrusion detection. *International Journal on Network Security*.

[9] A Ghaleb F., Saeed F., Al-Sarem M. (2020). Misbehavior-aware on-demand collaborative intrusion detection system using distributed ensemble learning for VANET. *Electronics*.

[10] Fu Y., Xiao N., Jiang H., Hu G., Chen W. (2019). Application-aware big data deduplication in cloud environment. *IEEE Transactions on Cloud Computing*.

[11] Sadotra P., Sharma C. (2017). A new distributed intrusion detection system in computer network: an approach to detect malicious intrusion threats at initial stage. *Oriental Journal of Computer Science and Technology*.

[12] Achbarou O., Kiram M. A. E., Elbouanani S., Xie Y. (2018). A new distributed intrusion detection system based on multi-agent system for cloud environment[J]. *International Journal of Communication Networks and Information Security*.

[13] Khonde S. R., Ulagamuthalvi V. (2019). Ensemble-based semi-supervised learning approach for a distributed intrusion detection system. *Journal of Cyber Security Technology*.

[14] Khonde S., Venugopal U. (2019). Hybrid architecture for distributed intrusion detection system. *Ingénierie des Systèmes d’Information*.

[15] Liu J., Zhang W., Ma T. (2020). Toward security monitoring of industrial Cyber-Physical systems via hierarchically distributed intrusion detection. *Expert Systems with Applications*.

[16] Singh S., Yassine A. (2018). Big data mining of energy time series for behavioral analytics and energy consumption forecasting. *Energies*.

[17] Yudong C., Yuejie C. (2018). Harnessing structures in big data via guaranteed low-rank matrix estimation[J]. *IEEE Signal Processing Magazine*.

[18] Yang K., Yu Q., Leng S. (2018). Data and energy integrated communication networks for wireless big data[J]. *Entia Sinica*.

[19] Jing Y., Bian Y., Hu Z., Wang L., Xie X.-Q. S. (2018). Deep learning for drug design: an artificial intelligence paradigm for drug discovery in the big data era. *The AAPS Journal*.

[20] Hong B., Wang H., Cao Z. (2021). An effective fault-tolerant intrusion detection system under distributed environment. *Wireless Communications and Mobile Computing*.

[21] Demertzis S., Demertzi V., Demertzis K. (2021). Data analytics for climate and atmospheric science. *International Journal of Big Data Mining for Global Warming*.

[22] Bian Y., Tang X. (2021). Abnormal detection in big data video with an improved autoencoder. *Computational Intelligence and Neuroscience*.

[23] Gattineni P., Dharan G. R. S. Intrusion Detection Mechanisms: SVM, random forest, and extreme learning machine (ELM).

[24] Cuzzocrea A. Big data lakes: models, frameworks, and techniques.

[25] Ra G., Kim T., Lee I. (2021). VAIM: verifiable anonymous identity management for human-centric security and privacy in the Internet of things. *IEEE Access*.

[26] Lijun Z., Guiqiu H., Qingsheng L., Guanhua D. (2021). An intuitionistic calculus to complex abnormal event recognition on data streams. *Security and Communication Networks*.

